# Fertility intentions and the way they change following birth- a prospective longitudinal study

**DOI:** 10.1186/s12884-020-02922-y

**Published:** 2020-04-17

**Authors:** Heidi Preis, Selen Tovim, Pnina Mor, Sorina Grisaru-Granovsky, Arnon Samueloff, Yael Benyamini

**Affiliations:** 1 Bob Shapell School of Social Work, Tel Aviv University, 69978 Tel Aviv, Israel; 2 Department of Psychology, Stony Brook University, Stony Brook, NY 11794-2500 USA; 3 Department of Obstetrics and Gynecology, Shaare Zedek Medical Center, affiliated with the Hebrew University Medical School of Jerusalem, Jerusalem, Israel

**Keywords:** Fertility intentions, Birth experience, Religiosity, Interpregnancy interval, Mode of delivery

## Abstract

**Background:**

Women’s fertility intentions, their desired number of children and desired inter-pregnancy interval (IPI) are related to micro (personal) and macro (socio-cultural) level factors. We investigated factors that contribute to changes in women’s fertility intentions in Israel, a developed country with high birth rates.

**Methods:**

Pregnant women (*N* = 1163), recruited from prenatal clinics and hospitals in two major metropolitan areas, completed self-report questionnaires prenatally (≥24 weeks gestation) and postpartum (2 months after childbirth). Women reported their socio-demographic background and obstetric history prenatally, their desired number of children and IPI at both time-points, and their objective and subjective birth experiences postpartum.

**Results:**

The findings indicated that background characteristics were related to prenatal fertility intentions. The strongest contributor to prenatal fertility intentions was women’s degree of religiosity- the more religious they were, the more children they desired and the shorter their intended IPI. Women’s postpartum fertility intentions were mostly consistent with their prenatal reports. In regression models, women who were very-religious, more educated and had previously given birth were less likely to report a lower number of desired of children at postpartum, compared to their prenatal report. Women who reported greater birth satisfaction and gave birth for the first time were less likely to change desired IPI.

**Conclusion:**

Having a negative birth experience could adversely affect women’s fertility intentions. Yet, in a pronatalist and medicalized birth culture, social pressures may decrease the effects of birth experiences on fertility intentions.

## Background

Fertility intentions, which are a component of fertility decision-making, are a major predictor of childbearing [1]. Fertility intentions have been defined in several ways, focusing on fertility desires, attitudes or behaviors [2]. In our current work, we conceptualize fertility intentions as the desire or intent to have a certain number of children and the desired or intended spacing between births. Women’s desires regarding having children are related to micro and macro level factors- the individual’s characteristics, personal unconscious motivations, and socio-cultural environment [3–6]. Less is known regarding the way experiences, especially the lived birth experience, relate to changes in fertility intentions. Quantitative and qualitative studies suggest that fertility intentions and fertility rates are related to women’s mode of delivery [7–9] and to the subjective experience of birth [10–12]. Yet, robust prospective studies documenting changes in fertility intentions following birth are rare [13]. The current study aims to investigate the contribution of the birth experiences and personal-cultural characteristics to changes in fertility intentions among women in Israel, a country with a unique reproductive context [14].

### Sociodemographic, cultural context and fertility intentions

Fertility intentions are related to both *tempo* intentions, the spacing between children or intra-pregnancy interval (IPI), and *quantum* intentions, the desired number of children [3]. Fertility intentions have been found to predict actual birth rates together with demographic and socioeconomic factors and cultural norms [15]. A comprehensive evaluation of changes in fertility intentions should account for these various socio-cultural factors.

Micro-level factors such as education, socioeconomic status, age, employment, sense of empowerment and household roles influence the decision to have additional children and the IPI [16–18]. In a representative study of the U.S. population, Cheslack-Postava & Winter [16] found that younger maternal age was associated with a longer IPI (greater than 60 months) and older maternal age (35 or older) with a shorter IPI (less than 12 months). The researchers interpreted the relationship between maternal age and a shorter IPI as a means to achieving the desired family size in a short amount of time. In a large study of European Union countries, there was a positive correlation between education and lifetime fertility intentions in 19 out of 27 countries. This correlation was significant in 7 of those countries. A significant negative correlation between education and fertility intentions was not found in any of the countries; in other words, evidence suggests that in some countries more educated women intend to have larger families [19].

Macro level factors such as societal normative expectations, religion, and policies affect fertility intentions and rates [4, 15, 20]. The current study was conducted in Israel, which is considered to be a modern yet traditional and pronatalist society [14]. Israel has the highest fertility rates in the Western industrialized world, with an average of 3.16 children per woman [21–23]. Despite declining fertility rates in the developed world in the last few decades, Israeli fertility rates remain high. Correspondingly, voluntary childlessness is rare and not a socially acceptable decision [24]. Women’s fertility intentions in Israel are formed vastly through tradition and gender roles, in addition to the predominant nationalistic and religious sentiments [14, 25]. Judaism, the dominant religion in Israel (74.3% of the population [26]), emphasizes motherhood and fertility as an important role for women: The religious commandment states that one should “be fruitful and multiply and replenish the earth” (Genesis 1:28) and fertility itself is considered a blessing (Deuteronomy 7: 13–14). The Muslim population in Israel is also characterized by high birth rates [27], but since the current research was conducted among mostly Jewish women, we will not elaborate on that here.

The Jewish population in Israel is made of several religious groups, which differ in their cultural beliefs, practices, and social norms. They could be broadly defined based on levels of observance of the Jewish traditions in their everyday life and social identity. Identification with a certain religiosity group impacts various life domains such as educational system choice, political ideology and day to day life such as dress code, prayer habits, and abstaining from activities on Saturday (the Jewish Holy day). The very-religious (Ultra-Orthodox) are the most observant group, followed by religious (Orthodox/Modern-Orthodox), traditional (slightly observant) and secular (non-observant Jews). The very-religious are the smallest group (approximately 10% of Jewish women 20 years of age and older), similar to the religious (11%), the traditional group (35%) is larger and the largest group are the secular (non-observant Jews), making up 44% [28]. As with other religions [29], in Judaism, fertility rates are influenced by religiosity [21, 25, 30]. Israeli very-religious women have high fertility rates, nearing 6.5 children per women. Fertility rates are lower among religious (4.5 children per woman), traditional (2.5 children per woman) or secular families (2.1 children per woman) [27]. Very-religious women often do not ‘plan’ the number of children they will have, adhering to the religious commandment that is interpreted as having as many children as God wills. Fertility issues (i.e. pregnancies, miscarriages, infertility, contraception) are considered a private matter [31] and thus it is likely that some very-religious women would not disclose specific fertility intentions (i.e., answering “do not know” or “whatever God chooses”). Nonetheless, research has shown that women who do not answer regarding fertility intentions in surveys are typically like most of their socio-cultural group [32].

### Reproductive history, birth experience and fertility intentions

Reproductive history such as parity, going through fertility treatments and perinatal experiences might affect women’s childbirth intentions [17]. The birth experience can be described in two different ways: the objective birth experience, referring to physical factors (e.g. cesarean delivery, vaginal assisted delivery, use of analgesia, home delivery) and the subjective birth experience, including personal emotions and attributions regarding the birth process (e.g. satisfaction with birth). Together and separately, these birth experiences are postulated to influence women’s intentions regarding future reproduction.

Mode of delivery is one of the most studied physiological factors in relation to subsequent childbirth. Operative deliveries, either cesarean delivery or instrumental vaginal delivery (such as vacuum extraction) may result in longer IPI compared to unassisted vaginal delivery [8, 9]. Subsequent deliveries have been found to decrease after a cesarean delivery compared to vaginal delivery [7, 33–35]. In addition, a decrease in the number of subsequent pregnancies was observed with respect to instrumental vaginal delivery, compared to unassisted vaginal delivery [9, 36]. One of the explanations of the effects of mode of delivery on subsequent childbirth is physiological and relates to the increased infertility risk following cesarean delivery [35, 37]. An alternative explanation focuses on the subjective experience of having a more traumatic birth. This is corroborated by findings from a qualitative study in which women conveyed their lack of interest for future pregnancies following an operative delivery, in spite of their previous large family plans [8]. An emergency operative delivery (whether vaginal instrumental or cesarean) could be perceived as a traumatic event, which increases childbirth-related fears and thus rendering women more reluctant to have another child [11, 38].

The subjective birth experience is related to cognitive, emotional and intra-personal factors such as, lack of control, fear or anxiety during birth, incongruence between expectation and reality in childbirth, and spouse or caregiver support [39, 40]. In a recent review [41], the detrimental effects of a negative birth experience were documented, however lack of rigorous empirical research on the subject was noticeable. Studies indicate that negative birth experiences decreased the chances of subsequent pregnancies and prolonged the IPI [11, 39]. Negative birth experiences and postpartum mental health difficulties were found to be related to a decrease in the number of subsequent pregnancies and increase in the IPI, regardless of the initial plan for family size [10, 42]. In contrast, satisfactory birth experiences, caregiver support, and positive emotions felt when seeing their baby for the first time, were among the factors that motivated women to give birth again [43, 44]. Concurrently, other studies did not find an indication that a negative birth experience affects women’s fertility intentions [41]. Therefore, there is a need for additional research that will assess the unique contributions of both the objective and subjective birth experiences and the socio-cultural context to changes in fertility intentions.

### Aims and hypotheses

There is a gap in knowledge related to the simultaneous and distinct effects of socio-cultural and reproductive factors on fertility intentions. The current study aimed to fill this gap by examining correlates of women’s fertility intentions (desired number of children and desired IPI) during pregnancy and following birth among a heterogeneous cohort of Jewish Israeli women. In the current study, we hypothesized that:

1. Individual factors such as lower religiosity, having had fertility treatments, and giving birth for the first time will be related to a change in fertility intentions in the postpartum period (desiring fewer children and/or increasing IPI). Other individual factors such as education and socioeconomic status will also be examined though there is insufficient basis to formulate clear hypotheses about the direction of these associations with fertility intention.
2. A more negative subjective birth experience and an emergency operative mode of delivery (instrumental vaginal delivery or emergency cesarean delivery) will be related to a change in fertility intentions in the postpartum period (desiring fewer children and/or increasing IPI).

## Methods

### Study design

The current investigation was a prospective longitudinal study. Eligibility criteria included being over 18 years of age and having a singleton pregnancy ≥24 weeks gestation with vaginal delivery medically possible. Exclusion criteria were pain (VAS > 3), medical emergency and non-Hebrew speaking. Participants completed questionnaires prenatally (T1 *M =* 32.38 ± 5.18 weeks gestation) and at 2 months postpartum (T2 *M =* 9.74 ± 2.37 weeks postpartum).

The study was approved by the Research Ethics Committees of all participating institutions. Recruitment took place between January 2016 and April 2018 at four Women’s Health Centers in the community and a hospital in the center of Israel (88.6% of participants), and another Hospital in Jerusalem (11.4% of participants). These locations service women from a variety of religious and non-religious communities.

### Procedures

Recruitment was conducted by a study team that included trained research assistants, social work graduate students and midwives. Women received an explanation about the study from a member of the study team and were asked for their written consent. Thereafter, they completed the first questionnaire (T1) using paper and pen in a quiet location at the clinic. Participation rates were 69.8%.

Follow-up was conducted by the study team approximately 2 months after the women’s due-date (T2). Women who provided their email address were sent a unique link to the questionnaire using Qualtrics software. Women who did not use email were mailed a paper questionnaire with a return envelope.

### Measures

***Socio-demographics and obstetric history*** were assessed at T1 and included basic socio-demographic questions such as age (in years), educational level (highest received degree which was coded as up to and including secondary education vs. higher education [academic degree]) and obstetric history questions such as whether they had previously given birth, if they had fertility treatments to conceive the current pregnancy and how many children they have. Women were asked to indicate their religion (Jewish, Muslim, Christian or Other) and religiosity group identification with the options commonly used in Israel (Secular, Traditional, Religious/Modern-Orthodox and Very-Religious/Ultra-Orthodox(. To gauge their socioeconomic status, women were asked how they perceive their financial status to be (below average, at average, or above average).

***Desired number of children*** were assessed at T1 and T2. Women were asked to indicate how many children they desire/ intend to have with the following options: 1 = One child, 2 = two children, 3 = three children, 4 = four children, 5 = five or more children, 6 = I do not know.

***Decrease in the desired number of children*** was calculated by subtracting T2 values of desired number of children from T1. Women who desired the same or a greater number of children between pregnancy and postpartum received a score of 0. Women who desired fewer children in the postpartum period compared to pregnancy were coded 1 (had a calculated T1-T2 value greater than 0).

***Desired IPI*** was assessed at T1 and T2. Women were asked “how many years after the upcoming/recent birth would you like to have another child?”. Besides a space to specify the number of years, women could choose the options “I will not want additional children” or “I do not know”.

***Increase in desired IPI*** was calculated by subtracting T1 IPI values from T2 values after excluding anyone who at T1 stated that they will not want additional children or those who answered “I do not know” at either timepoint. Women who reported a desired IPI of 6 months longer, the same IPI, or shorter in the postpartum measure compared to the measurement during pregnancy, received the score 0 indicating no increase in IPI. Women who reported a desired IPI of more than 6 months of the IPI reported during pregnancy, or changed their desire to not wanting additional children received a score of 1, indicating an increase in desired IPI.

***Emergency mode of delivery*** was assessed at T2 by asking where and how women gave birth, with the following options: Emergency cesarean delivery; planned cesarean delivery; instrumental vaginal delivery; vaginal delivery with epidural analgesia; vaginal delivery without epidural analgesia; vaginal delivery in a natural birth center; home birth. Women who gave birth either via emergency cesarean delivery or instrumental vaginal delivery received an emergency mode of delivery score of 1. The rest of the women received a 0, indicating not having an emergency delivery procedure.

***Birth satisfaction*** was assessed at T2 using the Childbirth Satisfaction Scale [45]. The scale includes 8 items assessing subjective general satisfaction with the birth experience. Women were asked to rate their agreement with the statements on a 1–5 scale. The measure was forward-and-back translated from English into Hebrew for the current study by two researchers who are fluent in both languages. It was then piloted with midwives and pregnant women who confirmed the face validity of the Hebrew version. The unidimensionality of the scale was confirmed via exploratory factor analysis and the reliability of the scale was high as determined by internal consistency (α = 0.93). Scores were computed as the average item response ranging from 1 to 5 with higher scores indicating greater satisfaction.

### Analysis

Analyses were performed using SPSS 24 [46]. At the first stage of the analysis, we describe the overall demographic, obstetric and prenatal fertility intentions using descriptive statistics, followed by univariate analyses of the associations between background variables and prenatal fertility intentions using χ^2^, independent samples *t* tests, Spearman’s Rho, and One-Way Analysis of Variance (ANOVA). In the final stage, we assessed the extent of changes in fertility intentions between the prenatal and postpartum measurement using paired *t* tests and χ^2^. Thereafter, we conducted multivariate logistic regressions with dichotomous dependent variables (no change vs. change in fertility intentions). Results were interpreted as significant at *p* < 0.05.

Missing value analyses indicated that there were very few missing variables (ranging 0.1–4.5%). Due to the small number of missing values, no data imputation was conducted; pairwise deletion was employed when possible and listwise deletion was used when pairwise was not possible. In the analyses of changes to desired number of children, very-religious women who answered “I do not know” about their desired number of children were regarded as desiring five or more children (since they are presumably similar to their peers [32]). Women who were not very-religious that answered “I do not know” were excluded from those analyses.

## Results

### Study population

Our current study included 1163 women who participated in two time-points of a large birth cohort study (1163 out of 1619 women who were recruited into the study at T1). Though follow-up rates were high (71.8%), there were significant differences in the main study variables between women who dropped-out of the study and those who were retained: Women who dropped out were more often very-religious, younger, less educated, had a lower socioeconomic status, had previously given birth, and desired to have more children, compared to women who did not drop-out.

Participants were on average 31.49 ± 4.64 years old. Almost a quarter of the sample were 35 or older. A little over half of the sample identified as secular, followed by traditional, religious/orthodox, and very-religious/ultra-orthodox. Most participants were married or cohabiting (98.0%, *n* = 1138) and were relatively educated, three-quarters of whom pursued academic, post-secondary education. Nearly half of the women reported that their socioeconomic status was on average. Over a third of the women were pregnant with their first child and the remainder were going to have their second to tenth child. A tenth of the women in the study used fertility treatments to achieve the current pregnancy (Table 1).

**Table 1:** Sociodemographic, obstetric history and fertility intentions of study participants (*N* = 1163)

	% (*n*)
**Religiosity**	
Secular	53.4 (619)
Traditional	19.0 (220)
Religious	15.9 (184)
Very-religious	11.7 (136)
**Socioeconomic status**	
Below average	11.9 (135)
Average	51.6 (584)
Above average	35.5 (413)
**Education level**	
No higher education	25.0 (289)
Higher education	75.0 (866)
**Maternal age**	
Younger (< 35)	76.4 (888)
Older (≥35)	23.6 (274)
**Fertility treatments**	
Spontaneous conception	89.3 (1028)
Had treatments	10.7 (123)
**Parity**	
First birth	40.7 (473)
Previously given birth	59.3 (688)
**Desired number of children**	
One	5 (0.4)
Two	157 (13.8)
Three	436 (38.7)
Four	239 (21.1)
Five or more	162 (14.3)
Does not know	133 (11.7)
**Desired Interpregnancy interval**	
1–1.5 years	150 (13.2)
2–2.5 years	372 (32.7)
3–3.5 yeas	237 (20.8)
4 years or more	46 (4.0)
No more children	95 (8.3)
Does not know	236 (21.0)

### Fertility intentions during pregnancy

Most of the women had decided prenatally on their desired number of children (88.3%, *n* = 1002) and reported a median of three desired children. Most of the women (91.7%, *n* = 1044) desired to have additional children after the upcoming birth. Of these women, 77.1% (*n* = 805) reported their desired IPI, which was on average 2.28 ± 0.85 years (Table 2).

**Table 2:** Fertility intentions during pregnancy among different religiosity groups (*N* = 1163)

	**Secular(*n*** **= 619)**	**Traditional** **(*n*** **= 220)**	**Religious** **(*n*** **= 184)**	**Very-religious** **(*n*** **= 136)**
**Desired number of children**				
One	5 (0.8)	0 (0.0)	0 (0.0)	0 (0.0)
Two	133 (21.7)	22 (10.1)	2 (1.1)	0 (0.0)
Three	336 (54.7)	87 (39.9)	14 (7.9)	1 (0.8)
Four	106 (17.3)	83 (38.1)	47 (26.4)	3 (2.5)
Five or more	5 (0.8)	8 (3.7)	83 (46.6)	65 (53.3)
Does not know	29 (4.7)	18 (8.3)	32 (18.0)	53 (43.4)
**Desired interpregnancy interval**				
1–1.5 years	66 (10.8)	35 (16.3)	26 (14.2)	21 (16.8)
2–2.5 years	211 (34.5)	73 (34.0)	56 (30.6)	30 (24.0)
3–3.5 yeas	135 (22.1)	48 (22.3)	40 (21.9)	14 (11.2)
4 years or more	31 (5.1)	6 (2.8)	6 (3.3)	3 (2.4)
No more children	74 (12.1)	14 (6.5)	5 (2.7)	2 (1.6)
Does not know	95 (15.5)	39 (18.1)	50 (27.3)	55 (44.0)

There were significant differences in the prenatal fertility intentions reported during pregnancy between women of different religiosity groups. Rates of not reporting fertility intentions, especially, desired number of children (answered “do not know”) grew with increasing religiosity. It is also noticeable that there was little variance in the desired number of children for very-religious women. Almost all (94.2%, *n* = 65) of those who reported, chose the option of “five or more children”. These findings support the assumption that women who did not report their desired number of children would have also answered “five or more children”, and thus, these women (*n* = 53) had their scores imputed for further analyses (Table 2).

Fertility intentions were related to all the socio-demographic and obstetric variables. One-way ANOVA of desired number of children indicated that the number increased linearly between the religiosity groups, with secular women desiring the least number of children, followed by traditional women, religious women and very-religious women who desired having the highest number of children. Post-hoc Scheffe tests revealed that each group significantly differed from the other. Regarding differences in desired IPI, ANOVA indicated that there were significant differences between the groups in their desired IPI. Yet, post-hoc Scheffe testing revealed that the only significant differences were between secular women, who desired a longer IPI, and the very-religious women, who desired the shortest IPI. Women with a higher education desired having fewer children and longer IPI compared to women who did not have more than secondary education. Women with higher socioeconomic status desired fewer children (*r* = − 0.22, *p* < 0.001) but this was unrelated to IPI (*r* = 0.05, *p* = 0.16). Women who had previously given birth desired more children and longer IPI compared to women who had not previously given birth. Having fertility treatment to achieve current pregnancy was related to fewer desired number of children and to a shorter desired IPI (Table 3).

**Table 3:** Associations between prenatal fertility intentions and women’s socio-demographic and obstetric background^†^

	*n*	Number of children^¥^		*n*	Interpregnancy interval	
		*M ± SD*	*t* / *F*		*M ± SD*	*t* / *F*
**Religiosity**			423.38^***^			4.07^**^
Secular	585	2.95 ± 0.69		443	2.37 ± 0.87	
Traditional	200	3.39 ± 0.73		162	2.20 ± 0.78	
Religious	146	4.45 ± 0.72		128	2.23 ± 0.82	
Very-religious	122	4.96 ± 0.24		68	2.04 ± 0.92	
**Education level**			2.90^**^			−2.14^*^
No higher education	253	3.77 ± 1.10		171	2.16 ± 0.89	
Higher education	796	3.38 ± 0.93		631	2.31 ± 0.84	
**Socioeconomic status**			29.72^***^			0.40
Below average	120	3.93 ± 1.07		96	2.17 ± 0.90	
Average	516	3.54 ± 1.01		397	2.29 ± 0.90	
Above average	396	3.22 ± 0.83		296	2.31 ± 0.78	
**Maternal age**			6.14^***^			4.56^***^
Younger (< 35)	810	3.57 ± 0.99		663	2.33 ± 0.84	
Older (≥35)	242	3.14 ± 0.94		142	2.02 ± 0.73	
**Parity**			−6.66^***^			−6.47^***^
First birth	436	3.24 ± 0.96		404	2.09 ± 0.74	
Previously given birth	576	3.64 ± 0.98		399	2.47 ± 0.92	
**Fertility treatments**			4.36^***^			3.77^***^
Spontaneous conception	929	3.52 ± 0.98		700	2.32 ± 0.85	
Had treatments	116	3.09 ± 0.99		95	1.97 ± 0.83	

^*^p < 0.05, ^**^p < 0.01, ^***^p < 0.001

^†^ These analyses excluded women who were not very-religious and answered “do not know” regarding desired number of children and all women who answered “do not know” or “do not want additional children” regarding desired IPI

^¥^Desired number of children were reported as 1–5 with 5 = five or more children

### The lived birth experience and changes in fertility intentions

There were 219 women (18.8%) who had an emergency operative delivery (i.e. instrumental vaginal delivery [9.7%, *n* = 113] or emergency cesarean delivery [9.1%, *n* = 106]). The remainder of the participants had a vaginal delivery with epidural analgesia (46.6%, *n* = 541), vaginal delivery without using analgesia (26.9%, *n* = 312), and planned cesarean delivery (7.7%, *n* = 89). Average satisfaction with childbirth was 3.89 ± 1.03. Having an emergency delivery was significantly associated with lower levels of birth satisfaction (2.89 ± 1.05) compared to women who did not have an emergency delivery (4.12 ± 0.87) (*t*(288) = 15.96, *p* < 0.001).

Women were mostly consistent in their fertility intentions when comparing prenatal and postpartum responses: the desired number of children and desired IPI reported at T1 were highly correlated with the T2 reports (*r* = 0.88 and *r* = 0.63, *p*s < 0.001 respectively). Paired sample *t*-test, indicated a slight significant decrease in women’s desired number of children between T1 (3.48 ± 0.99) and T2 (3.44 ± 1.01) (*t*(1006) = 2.22, *p* = 0.03). There was also a slight significant overall decrease in desired IPI between T1 (2.27 ± 0.85) and T2 (2.18 ± 0.92) (*t*(730) = 3.40, *p* = 0.001). There were 98 (9.7%) women who reported desiring fewer children at T2 than they did at T1 (decreased their desired number of children). There were 111 (14.8%) women who reported desiring a longer IPI at T2 compared to T1 (increased their desired IPI) or changed their minds from desiring additional children to not desiring additional children. Women who decreased their desired number of children were twice as likely to also increase their desired IPI compared to women who did not decrease their number of desired children (χ^2^(1) = 9.01, *p* = 0.003).

Multivariate logistic regressions indicated that some of the study variables were uniquely predictive of a change in fertility intentions (decrease in desired number of children vs. no decrease and increase in desired IPI vs. no increase in desired IPI). The first model indicated that being very-religious, having a higher education and having previously given birth were all uniquely associated with a lower likelihood to desire fewer children in the postpartum period, compared to the prenatal period. The birth experiences (having an emergency delivery, birth satisfaction), age, fertility treatments, and socioeconomic status were unrelated to changes in the desired number of children. In the model predicting change in IPI, lower birth satisfaction and having previously given birth were the only predictors uniquely associated with a greater likelihood of increasing desired IPI in the postpartum period (Table 4).

**Table 4:** Logistic regression predicting changes in fertility intentions

	No decrease in desired number of children vs. decrease in desired number of children *n* = 971			No increase in desired IPI vs. increase in desired IPI *n* = 728		
	B	OR	95% CI	B	OR	95% CI
Religiosity	−2.77	0.06^**^	0.01–0.47	0.03	1.03	0.48–2.20
Education	−0.85	0.43^**^	0.26–0.71	−0.42	0.66	0.40–1.09
**Socioeconomic status**	−0.01	0.99	0.72–1.36	−0.07	0.93	0.69–1.25
Age	0.12	1.13	0.65–1.96	0.21	1.23	0.71–2.14
Parity	−0.72	0.49^**^	0.30–0.80	0.55	1.73^*^	1.10–2.72
Fertility treatment	−0.06	0.87	0.48–1.86	−0.57	0.56	0.26–1.23
Emergency delivery	0.12	1.13	0.62–2.07	−0.29	0.75	0.41–1.37
Birth satisfaction	−0.20	0.82	0.65–1.04	−0.46	0.63^***^	0.51–0.79

*Note:* These analyses excluded women who were not very-religious and answered “do not know” regarding desired number of children at either time point and any women who answered “do not know” regarding desired IPI or that did not want additional children at T1. Religiosity (not very-religious = 0, very-religious = 1); Education (no higher education = 0, higher education = 1); Age (less than 35 years old = 0, 35 years old or older = 1); Parity (first birth = 0, previously birthed = 1); Fertility treatments (spontaneous conception = 0, had fertility treatments = 1); Emergency delivery (unassisted vaginal delivery or planned cesarean delivery = 0, assisted vaginal delivery or emergency cesarean delivery = 1)

^*^p < 0.05, ^**^p < 0.01, ^***^p < 0.001

In order to assess the moderating effect of religiosity on changes in fertility intentions, we also ran the regression models separately for very-religious women and the significant predictors stayed the same in all the models, suggesting no interaction.

## Discussion

Findings of this investigation of prenatal and postpartum fertility intentions confirmed the hypothesis regarding the importance of socio-demographics (especially religiosity) and obstetric history for quantum and tempo fertility intentions. These factors explained more of the variance in desired number of children than they did in the desired IPI. The hypothesis regarding the impact of childbirth experiences on fertility intentions was only partially confirmed. In multivariate analyses, satisfaction was significantly related only to an increase in IPI and an emergency mode of delivery was unrelated to fertility intention changes.

The social norms regarding the importance of motherhood in the Jewish Israeli society [14, 24] were also reflected in our findings. Almost all the women in our study (99.5%) desired to have more than one child and of the women who desired additional children, two-thirds (64.8%) desired a relatively short IPI of 2.5 years or less. Religiosity was the main predictor of prenatal fertility intentions. This is similar to findings from other national studies, which indicate that very-religious women have the highest fertility rates, followed by religious women, traditional women and secular women [25, 27, 30]. It is not surprising that almost all the very-religious women in our study reported desiring five children or more. This was strengthened by the finding that among the very-religious women, there was hardly any change to the postpartum desired number of children. Bearing children is the fulfillment of womanhood in the religious society and is a central life goal for religious women [31]. Raising a large family is held in high esteem and contributes to the woman’s happiness and self-fulfillment, while not having children, causes great distress [47].

Contrary to our hypothesis, the effect of the objective birth experience on changes in fertility intentions was not significant after controlling for socio-demographic factors and the subjective birth experience. This is similar to other studies that did not find an association between mode of delivery and fewer subsequent pregnancies [36, 48]. Since in Israel, as in most of the Western world, birth is medicalized, it is possible that having an emergency mode of delivery is not viewed as outside of the norm [49]. While the physical experience of having a medicalized birth is acceptable, the subjective experience, which might be overwhelming, may consequently affects women’s future fertility [50].

In our study, when we examined the unique contribution of birth satisfaction using regression models, it was only related to the increase in desired IPI and not to the desired number of children. It is possible that the timing of the next pregnancy is more flexible and it is likely that women who have had a dissatisfying birth might want to delay having another child until they felt more emotionally ready. On the other hand, the desired number of children is a more rigid fertility intention, particularly in a pronatalist culture, where even having only one child is unacceptable and stressful for women [51]. Social pressure on reproduction is particularly strong in the religious population, which is reflected in the strong associations of religiosity and fertility intentions in our sample. In this socio-cultural context, it is unlikely that a personal experience such as a negative subjective birth experience is enough to change such a fundamental planned course of life as the number of children, though as seen here, it could extend the IPI. In a small Israeli study, postpartum post-traumatic stress was related to not wanting to have additional children when women were questioned shortly after giving birth [42]. Conceivably, in a pronatalist culture, only in extreme cases where the birth experience creates lasting or severe mood disturbances, a major fertility intention such as the desired number of children is affected.

Parity was a strong predictor of fertility intentions and their change postpartum, but in different ways. First-time mothers were more likely to decrease their desired number of children but less likely to change their desired IPI. It is possible that for first-time mothers, the transition to motherhood is more dramatic and the reality and difficulties that accompany caring for a child, especially in the first few months of their lives, bring some women to change their mind regarding the desired number of children. The adjustment to having (another) child is less difficult for experienced mothers and thus less likely to affect the desired number of children. Another possible explanation is that first-time mothers in a pronatalist country are motivated to proceed with expanding their family to the least acceptable family size (two children) and are therefore less likely to increase IPI following birth. It is possible that since experienced mothers already have a larger family, they are more flexible regarding their desired IPI compared to first-time mothers. This is also reflected in the prenatal differences in desired IPI which were longer for experienced mothers. Another possible explanation is that having a second or more child makes women feel more familial fulfillment or more exhaustion and thus they would be more inclined to postpone their next birth a little longer. Finally, women who have obtained post-secondary higher education (i.e., university degree) were less likely to decrease their desired number of children. This finding might be explained in light of a recent meta-analysis of European fertility intention studies which found a positive association between education and intentions to reach normative family size [52]. Another explanation to these findings may be related to the greater human capital that more educated women may have, which might help them preserve their initial fertility intentions when faced with the reality and burden which might be associated with having a child. At the same time, as opposed to other developed European countries [19], in our sample there was a negative association between education and prenatal fertility intentions, which was most likely due to the confounding of education and religiosity levels. Therefore, the association between education and fertility intentions, which is a complex association, beyond the scope of our research, should be interpreted with caution.

### Strengths and limitations

The main strengths of our investigation were its prospective design and the large sample of women with varying religious backgrounds. Studies in the field of fertility intention changes are few and limited and so are studies which examine the effects of the birth experience on fertility intentions [41]. By conducting a longitudinal study, we were able to rigorously assess changes in two types of fertility intentions. Moreover, we were able to examine the effects of both the objective and subjective birth experiences in addition to other contributing factors. The high fertility rates in the Israeli population make it an ideal context to study changes in fertility intentions following childbirth. The higher fertility rates allow more room for change in fertility intentions and diminish floor and ceiling affects which may persist in countries where women ordinarily have one or two children. The heterogenous sample, in terms of religiosity levels, allowed for a more sensitive investigation since religiosity may play such an important role in women’s fertility intentions.

Our investigation did have some limitations. It did not account for additional variables which could affect women’s fertility intentions such as the infant’s temperament, which has been found to affect planned and actualized family size [39]. The generalizability of our study was limited to the Israeli Jewish urban population. Women in the very-religious group had high rates of not reporting fertility intentions. This inclination not to answer is mostly likely due to religious reasons or privacy concerns [31] and does not reflect different intentions [32], thus we imputed data for these women, under the assumption that they would have answered wanting five children or more like the rest of their religiosity group. Even though analyses without this imputation showed similar results (not presented), this procedure should be noted. There were differences between women who dropped out compared to those who did not in most of the study variables. It is likely that most of these differences were because the very-religious women in the study were mostly followed-up by a paper questionnaire sent by mail compared to the rest of the women who had internet access and completed the T2 questionnaire online. This methodological barrier could have introduced bias to our findings, though probably in the form of underestimation. Finally, we asked women regarding their fertility intentions 2 months postpartum. Despite the methodological and theoretical justification of assessing birth experience at that time point [53, 54] it is possible that with time, women will go back to their initial fertility intentions. Nonetheless, it does not take away from the importance of the findings- a negative birth experience has strong implications on women lives, at least in the first few months postpartum when women must take on the motherhood role and function.

## Conclusion

It is possible that in a pronatalist country such as Israel, women’s fertility intentions are not easily modified. When social norms, public policy and religious beliefs pressure women to bear children [24], it is less likely that the birth experiences would affect women’s childbearing intentions. In other words, given these social pressures, women have less freedom to cope with negative birth experiences by changing their fertility plans; they might feel pressure to have another child, even when they are not ready for it. This pressure to conceive is especially strong among very-religious women who speak less about their difficulties and utilize birth spacing methods less often [31]. Mistimed pregnancies, or pregnancies following a negative birth experience can elicit additional distress. Therefore, it is important to detect these difficulties and address them to improve well-being in subsequent pregnancies and satisfaction in future childbirths.

Future studies should examine actual subsequent births, their timing and their effects on maternal adjustment among different populations. Relinquished fertility could cause great distress [39] and it is important to understand why it might happen. More studies need to examine the role of the subjective birth experience in changes to fertility plans, especially in countries where fertility rates are lower and social pressures on having (additional) children are minor. In such countries, the negative effects the birth experience might have on fertility intentions could go unnoticed and potentially hinder attempts to increase birth rates. This could also help in developing appropriate interventions to ameliorate the deleterious effects of a negatively experienced birth.

## Data Availability

The datasets used and/or analyzed during the current study are available from the corresponding author on reasonable request.

## Abbreviation

IPI: Interpregnancy interval
